# Supplementary material for: Quantum fluids of light in all-optical scatterer lattices
Source: Nat Commun. 2021 Sep 22;12:5571. doi: 10.1038/s41467-021-25845-4 (PMC8458361; doi:10.1038/s41467-021-25845-4)
Supplement: Supplementary file 1 — Supplementary Information [file 41467_2021_25845_MOESM1_ESM.pdf]

Supplementary Information: Quantum fluids of light in all-optical scatterer lattices

S. Alyatkin,[1,2] H. Sigurdsson,[1,2,3] A. Askitopoulos,[1,2] J. D. Töpfer,[1,2] and P. G. Lagoudakis[1,2,3,*]

[1]Center for Photonics and Quantum Materials, Skolkovo Institute of Science and Technology, Moscow, Russia
[2]Laboratories for Hybrid Photonics, Skolkovo Institute of Science and Technology, Moscow, Russia
[3]School of Physics and Astronomy, University of Southampton, Southampton, SO17 1BJ, United Kingdom

(Dated: August 25, 2021)

SUPPLEMENTARY NOTE 1: ADDITIONAL EXPERIMENTAL MEASUREMENTS ON OPTICAL LATTICES WITH SQUARE, LIEB, AND INVERSE LIEB GEOMETRY

In this section we provide additional data on the measured polariton photoluminescence (PL) in the diffractive scatterer regime using pump spots arranged in a square lattice with lattice constant $D = 22.5$ µm (see Supplementary Fig. 1) and Lieb lattice $D = 22.3$ µm (see Supplementary Fig. 2). We also present complementary data on the inverse Lieb lattice in Fig. 4 in the main manuscript (see Supplementary Fig. 3). The full-width-half-maximum (FWHM) of the Gaussian laser spots exciting the condensates is ≈ 2 µm. Just like in the main text, we define the real space horizontal and vertical coordinates as $\mathbf{r} = (x, y)$, and in momentum space $\mathbf{k} = (k_x, k_y)$.

Supplementary Figs. 1a and 1b show the PL in real space and energy-momentum space (along $k_x = 0$) approximately at threshold ($P \approx P_{\text{thr}}$) for a square arranged lattice of widely spaced Gaussian pump spots. At this power no significant phase locking occurs between the condensates as can be evidenced from the absence of interference fringes in the spatial PL and the broad energy distribution of polaritons in the lattice dispersion. In Supplementary Figs. 1c and 1d we show the same measurements above condensation threshold ($P \approx 1.3P_{\text{thr}}$) which result now in clear appearance of interference fringes between the radiating condensates and a greatly narrowed energy distribution peaking around 1.4478 eV. We note that Supplementary Figs. 1b and 1d are plotted on a logarithmic scale. We observe multiple illuminated high energy bands in the polariton dispersion verifying that polaritons in the system are experiencing crystal scattering in the optical lattice.

In Supplementary Fig. 2 we provide similar measurements to those in Fig. 2a-e in the main manuscript but using

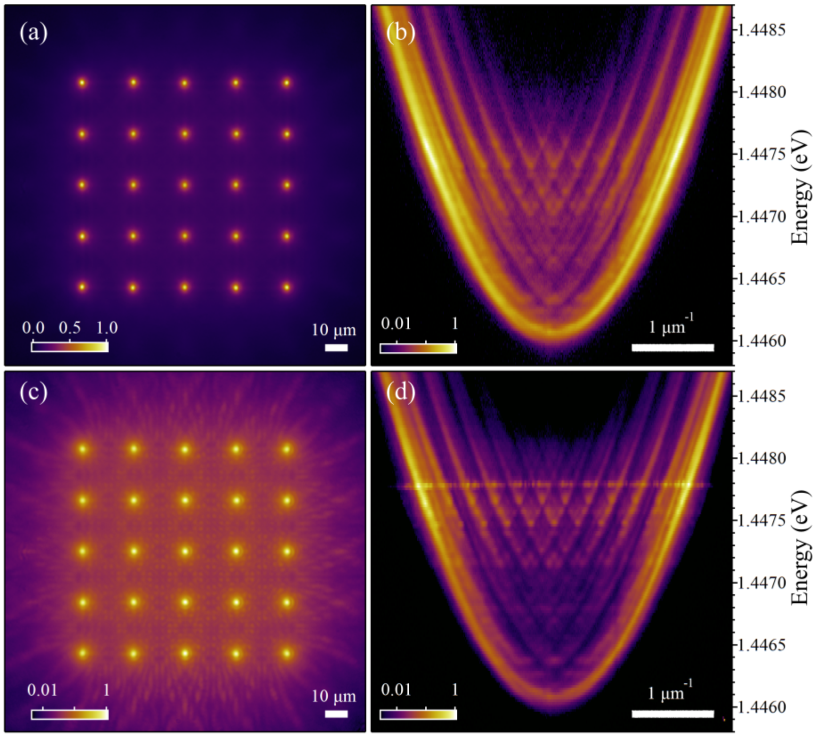

Supplementary Figure 1. **Optical lattice of 25 polariton condensates arranged in a square geometry** with a lattice constant set to $D = 22.5$ µm. (a,c) Real space and (b,d) energy-resolved momentum space polariton PL at ($P \approx P_{\text{thr}}$) and above ($P \approx 1.3P_{\text{thr}}$) threshold respectively.

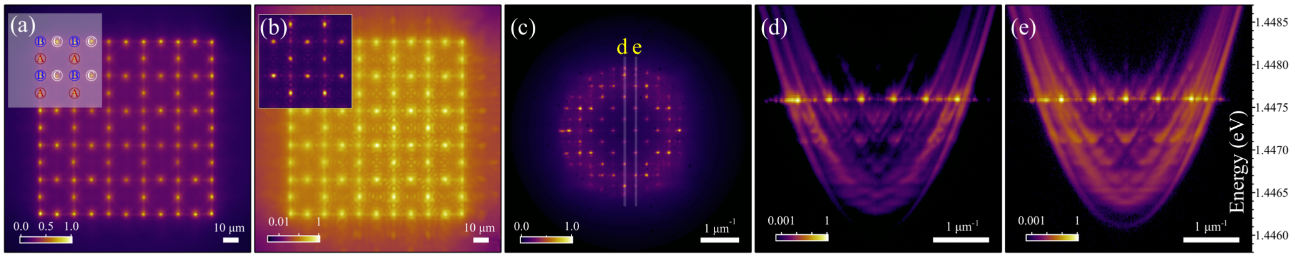

Supplementary Figure 2. **Optical lattice of 96 polariton condensates arranged in a Lieb geometry** (a) at threshold with a lattice constant set to $D = 22.3$ μm. The inset in (a) schematically shows sublattices denoted with letters A,B,C forming the Lieb lattice. (b) Real space polariton PL for the lattice pumped at $P \approx 1.1 P_{\text{thr}}$. Driving the system above threshold leads to the condensation on A and C sublattices, see inset shown in linear colorscale. (c) Momentum space polariton PL for $P \approx 1.1 P_{\text{thr}}$. Semi-transparent thin lines denoted with yellow letters "d" and "e" correspond to $k_x = 0$ and $k_x = 2\pi/D$, for which the energy-resolved momentum space PL shown in (d),(e) respectively.

a larger lattice constant of $D = 22.3$ μm. The results presented in Supplementary Fig. 2 show that by changing the lattice constant in the scatterer regime we can change the qualitative features of the polariton PL above threshold. Here we observe destructive interference inside the cells of the lattice (i.e., where pump spots are absent) whereas in Fig. 2 (main manuscript) we saw constructive interference. This result can be intuitively understood from the fact that polaritons are condensing into a favorable Bloch mode of the optical lattice by maximising their mutual interference (overlap) over the pump gain region. Changing the lattice constant D naturally affect the interference (i.e., more wavelengths fit between pump spots) and one observes distance-periodic changes in the polariton PL pattern as different Bloch modes move in and out of the lattice gain bandwidth.

In Supplementary Fig. 3 we provide experimental results for the inverse (conventional tight binding) Lieb lattice hosting P-flatbands corresponding to Figs. 4e-g in the main text. Supplementary Fig. 3a shows the energy-resolved reciprocal space PL taken at $k_x = 0$ where yellow arrows indicate the location of P- and S-bands. Supplementary Fig. 3b is measured at $k_x = 2\pi/D$ where the two horizontally dashed lines, denoted with yellow letters "(c)" and "(d)" correspond to the momentum space isoenergy planes PL shown in Supplementary Figs. 3c and 3d. We note that in each panel the PL is independently normalised. The data shown in Supplementary Fig. 3c is the same as shown in Fig. 4l in the main manuscript and corresponds to polariton condensation into the M symmetry point of the P-flatband. Unfortunately, the limited energy resolution of our experimental setup and the small non-stimulated population of polaritons in surrounding band structures prevents us from accurately resolving the P-flatbands in energy-momentum space. We additionally present the momentum space PL corresponding to the lowest P-band, forming an peculiar square shape pattern along the $\Gamma \leftrightarrow X$ edges in the outer Brillouin zones. The lowest P-band is completely flat along the $\Gamma \leftrightarrow X$ edges but possesses strong dispersion along the $X \leftrightarrow M \leftrightarrow \Gamma$ edges as shown in Fig. 4i in the main manuscript. The same type of PL is also observed in our calculations (see Supplementary Fig. 7e).

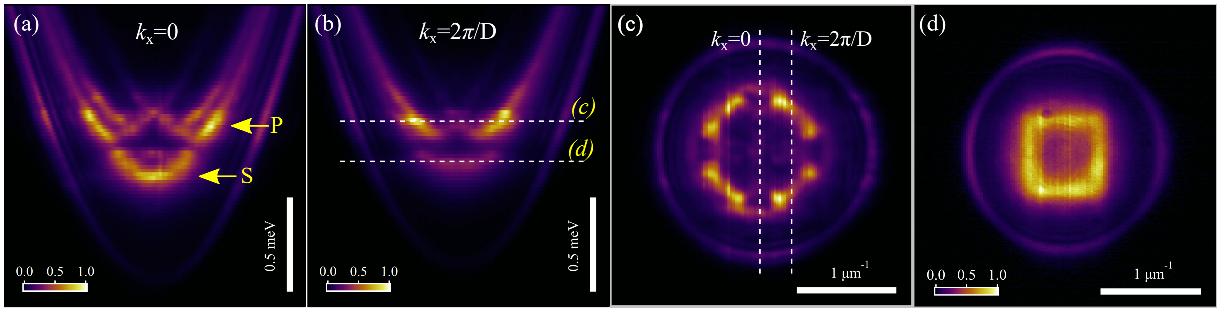

Supplementary Figure 3. **Experimentally measured band structure in an inverse Lieb lattice with $D = 16$ μm.** (a,b) Energy-resolved momentum space polariton PL at $k_x = 0$ and $k_x = 2\pi/D$ corresponding to the system shown in Figs. 4e-g of the main text. Yellow arrows in (a) indicate P-band and S-band states respectively. White dashed lines in (b) with yellow letters "(c)" and "(d)" specify the energies of the momentum space PL presented in (c) and (d), respectively. All panels are normalised independently.

SUPPLEMENTARY NOTE 2: THEORY OF POLARITON DYNAMICS

We consider the two-dimensional (2D) Schrödinger equation which describes a polariton in the static potential landscape designed by our optical non-resonant lasers.

$$i\hbar\frac{d\Psi}{dt} = \left[\hat{E} + V(\mathbf{r})\right]\Psi \tag{1}$$

Here, $\hat{E}$ is the dispersion of the lower polariton branch. The upper polariton branch is neglected because it is both far away in energy and weakly populated by our experimental excitation scheme. Given the much larger effective mass of the excitons compared to the cavity photons we can set their dispersion to zero and the lower polariton dispersion in momentum space becomes[1],

$$\hat{E}(\mathbf{k}) = \frac{1}{2}\left[\hat{E}_\phi(\mathbf{k}) - \sqrt{\hat{E}_\phi^2(\mathbf{k}) + (\hbar\Omega)^2}\right], \tag{2}$$

where $\hat{E}_\phi(\mathbf{k})$ is the cavity photon dispersion and $\hbar\Omega = 12$ meV is the Rabi energy of our sample[2]. The longitudinally confined photons obey the dispersion relation,

$$\hat{E}_\phi(\mathbf{k}) = \sqrt{\left(\frac{hc}{\lambda}\right)^2 + \left(\frac{\hbar ck}{n}\right)^2} + \Delta - \frac{i\hbar\gamma}{2}, \tag{3}$$

where $\lambda = 860$ nm is the cavity resonance, $n = 3.556$ is the cavity refractive index, $\Delta = -5$ meV is the cavity photon detuning from the exciton resonance, and $\gamma = 1/5.5$ ps^{-1} is the cavity photon lifetime. The real part of the calculated dispersion is shown with a white dashed line plotted against the experimentally retrieved dispersion in Supplementary Fig. 4a. In Supplementary Fig. 4b we show a schematic of the Lieb lattice and the three square sublattices denoted by their label A, B, C.

The potential $V(\mathbf{r})$ is directly proportional to the incident non-resonant laser intensity and can be written as a superposition of spatially separated Gaussians,

$$V(\mathbf{r}) = V_0 \sum_{n=1}^{N} \exp\left[-\frac{(x-x_n)^2 + (y-y_n)^2}{2w^2}\right]. \tag{4}$$

Here, N is the number of pumps in question and x_n, y_n are their coordinates, V_0 denotes the complex potential strength. $\mathrm{Re}(V_0) > 0$ and $\mathrm{Im}(V_0) > 0$ denote the blueshift and particle gain experienced by polaritons respectively due to their interactions with the laser generated background of non-condensed particles, and $w = 1.274$ μm is the RMS width of the pump spots corresponding to 3 μm FWHM. The width is slightly larger than that of the lasers (2 μm FWHM) to account for the finite diffusion of excitons in the reservoir from the laser spot.

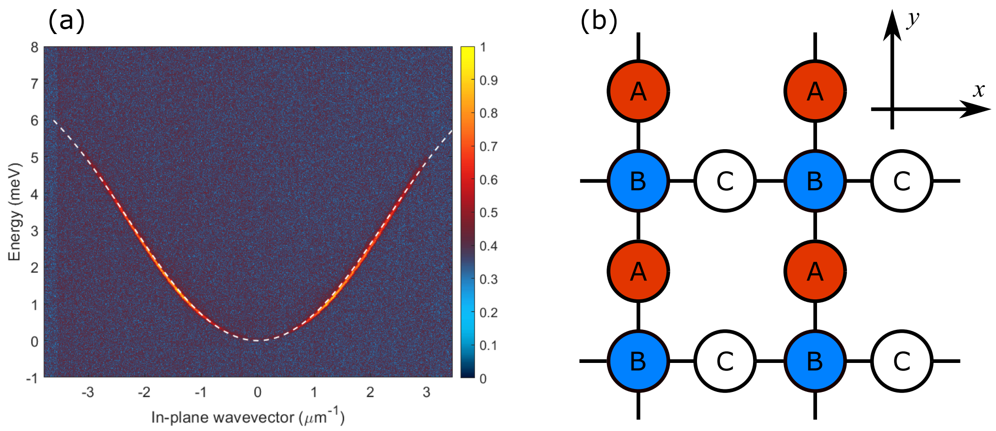

Supplementary Figure 4. **Bare lower polariton dispersion and Lieb lattice illustration.** (a) Heatmap shows the normalized measured lower polariton dispersion with Eq. (2) plotted on top as a white dashed line. (b) Schematic of the Lieb lattice geometry consisting of three square sublattices labeled A, B, C.

The dispersion of Eq. (1) can be numerically obtained through Monte-Carlo methods where an average is performed over the time evolution of many random initial realizations of the particle wavefunction $\Psi(\mathbf{r}, t = 0)$. This method is particularly useful for potentials which lack the usual symmetries required by Bloch's theorem to work. It permits calculation over which frequency and momentum components $\tilde{\psi}(\omega, \mathbf{k})$ survive the longest in the system (i.e., decay the slowest) in presence of optical gain at the pump spots [i.e., $\text{Im}(V_0) > 0$]. Results of this method are shown in Supplementary Figs. 5-7 where we extract numerically the dispersion from the dynamics of many random initial realizations of $\tilde{\psi}(\omega, \mathbf{k})$ in Eq. (1) in several lattice geometries relevant to the study. Overall we find good agreement between experimental observations and our calculations across all lattice geometries.

SUPPLEMENTARY NOTE 3: BLOCH THEOREM

In this section we investigate the dispersion belonging to Eq. (1) by applying Bloch's theorem. The following equations were used to calculate the bands plotted in Figs. 2i and 4i in the main text. For simplicity, we apply a parabolic approximation by writing,

$$\hat{E}(\mathbf{k}) = \frac{\hbar^2 k^2}{2m} \tag{5}$$

where the effective mass of the lower polaritons is $m = 0.32$ meV ps^2 μm^{-2} found by fitting the dispersion in Supplementary Fig. 4a. The potential is assumed to be infinite and periodic such that $V(\mathbf{r}) = V(\mathbf{r} + \mathbf{D})$ where $\mathbf{D} = n_1\mathbf{a}_1 + n_2\mathbf{a}_2$ is the translational symmetry vector defined in the bases of primitive lattice vectors $\mathbf{a}_{1,2}$ for some integers $n_{1,2}$. We can then apply Bloch's theorem where we write the polariton single particle wavefunction in the factorised form of crystal momentum $\mathbf{q} = (q_x, q_y)$ and Bloch states in the nth band $u_{n,\mathbf{q}}(\mathbf{r})$,

$$\Psi_n(\mathbf{r}) = e^{i\mathbf{q}\cdot\mathbf{r}} u_{n,\mathbf{q}}(\mathbf{r}). \tag{6}$$

Here, $u_{n,\mathbf{q}}(\mathbf{r}) = u_{n,\mathbf{q}}(\mathbf{r} + \mathbf{D})$. Substituting Eq. (6) into Eq. (1) the Bloch eigenvalue problem then reads,

$$\left[\frac{\hbar^2}{2m}\left[\left(q_x - i\frac{\partial}{\partial x}\right)^2 + \left(q_y - i\frac{\partial}{\partial y}\right)^2\right] + V(\mathbf{r})\right] u_{n,\mathbf{q}}(\mathbf{r}) = E_{n,\mathbf{q}} u_{n,\mathbf{q}}(\mathbf{r}). \tag{7}$$

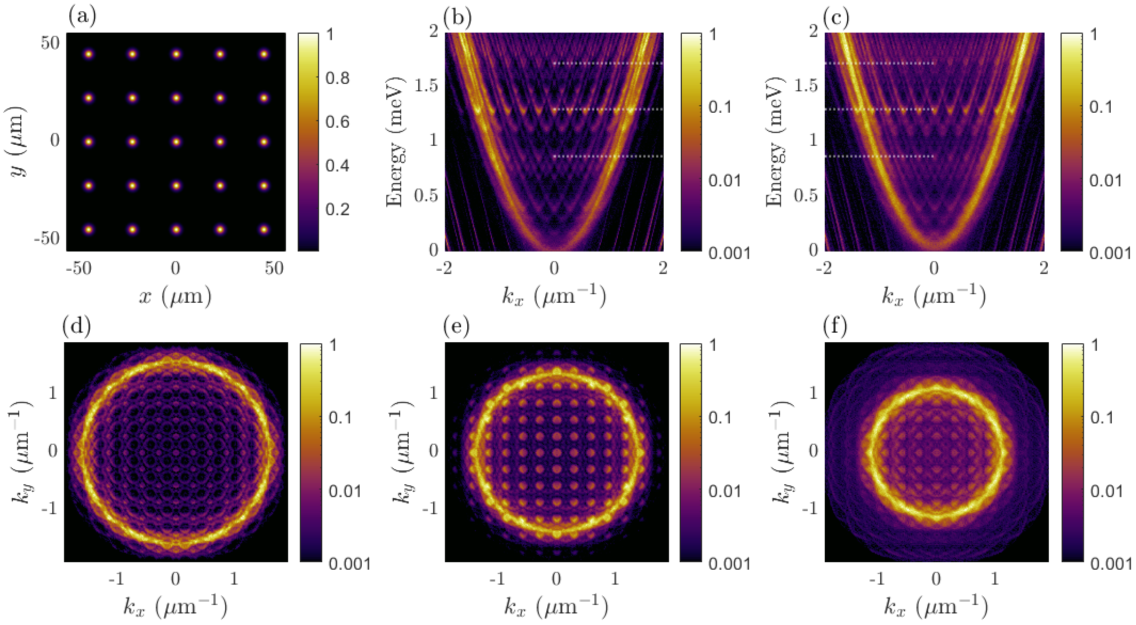

Supplementary Figure 5. **Calculated band structure in a square arranged scatterer lattice of Gaussian spots.** (a) Non-resonant laser profile $V(\mathbf{r})$ (normalised) shaped into a square scatterer lattice of Gaussians with a lattice constant $D = 22.5$ μm and $V_0 = 2 + 0.25i$ meV. (b,c) Energy-momentum space PL at (b) $k_y = 0$ and (c) $k_y = 2\pi/D$. (d-f) Polariton momentum space PL around threshold at $E = 1.72, 1.3, 0.87$ meV respectively corresponding to the white dotted lines in (b,c).

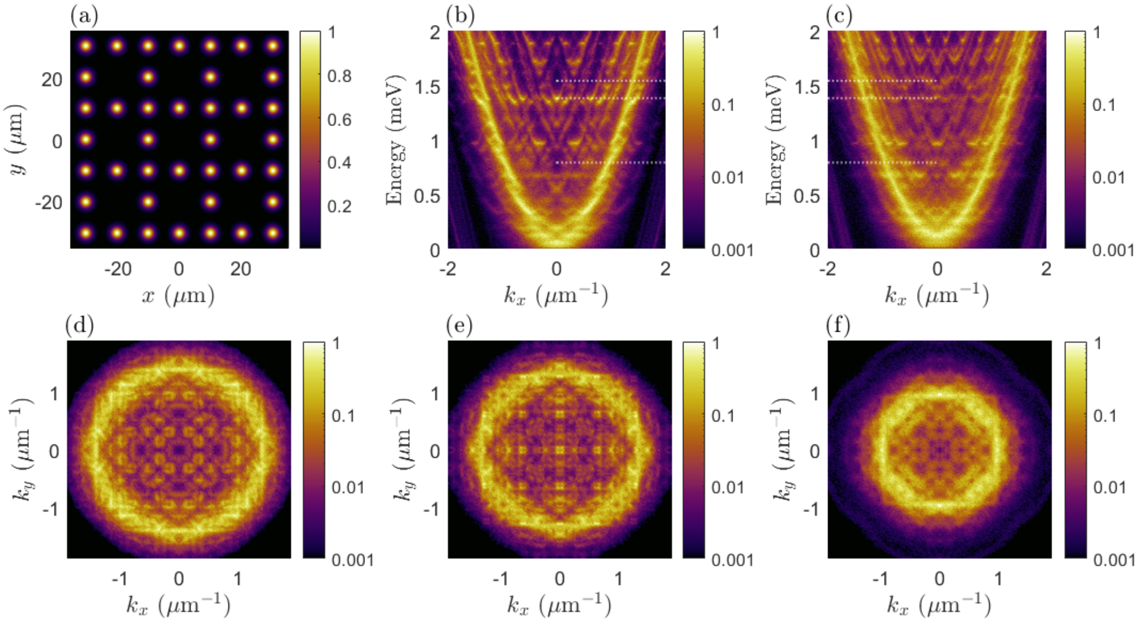

Supplementary Figure 6. **Calculated band structure in a Lieb arranged scatterer lattice of Gaussian spots.** (a) Non-resonant laser profile $V(\mathbf{r})$ (normalised) shaped into a Lieb scatterer lattice of Gaussians with a lattice constant $D = 20.3$ µm and $V_0 = 2.2 + 0.25i$ meV corresponding to Fig. 2 in the main text. (b,c) Energy-momentum space PL around threshold for (b) $k_y = 0$ and (c) $k_y = 2\pi/D$. (d-f) Polariton momentum space PL around threshold at $E = 1.54, 1.38, 0.79$ meV respectively corresponding to the dotted lines in (b,c).

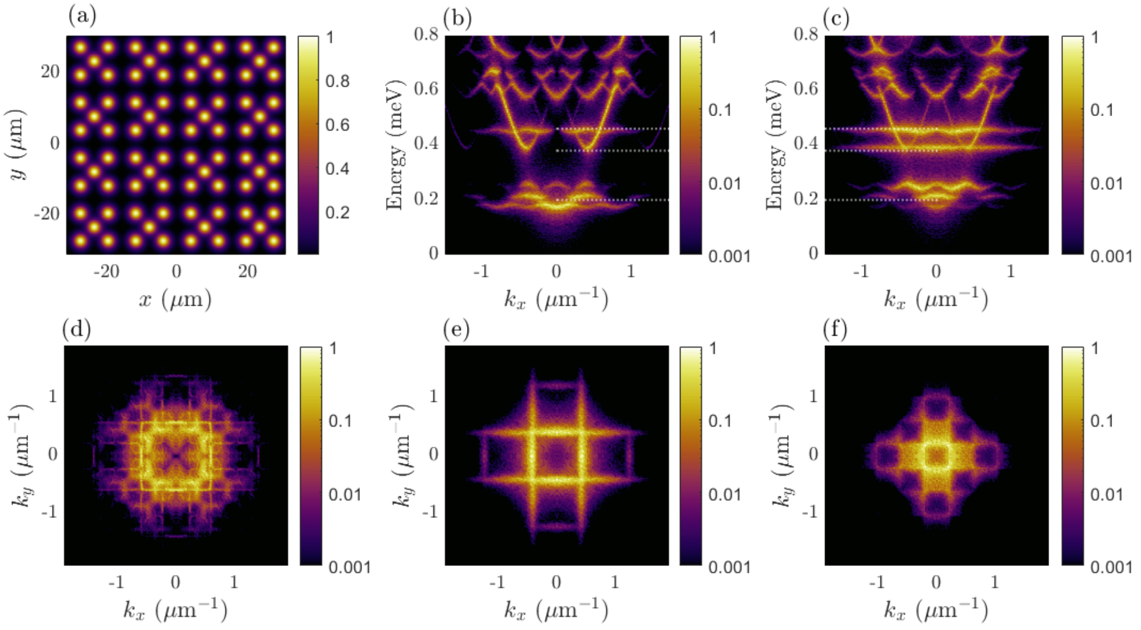

Supplementary Figure 7. **Calculated band structure in a Lieb lattice formed from inverse arrangement of Gaussian pump spots.** (a) Non-resonant laser profile shaped to form a conventional Lieb lattice of potential minima between the lasers. Lattice constant is $D = 15.6$ µm and $V_0 = 2$ meV corresponding to Fig. 4 in the main text. (b,c) Energy-momentum space PL around threshold for (b) $k_y = 0$ and (c) $k_y = 2\pi/D$. (d-f) Polariton momentum space PL around threshold at $E = 0.47, 0.38, 0.2$ meV respectively corresponding to the dotted lines in (b,c).

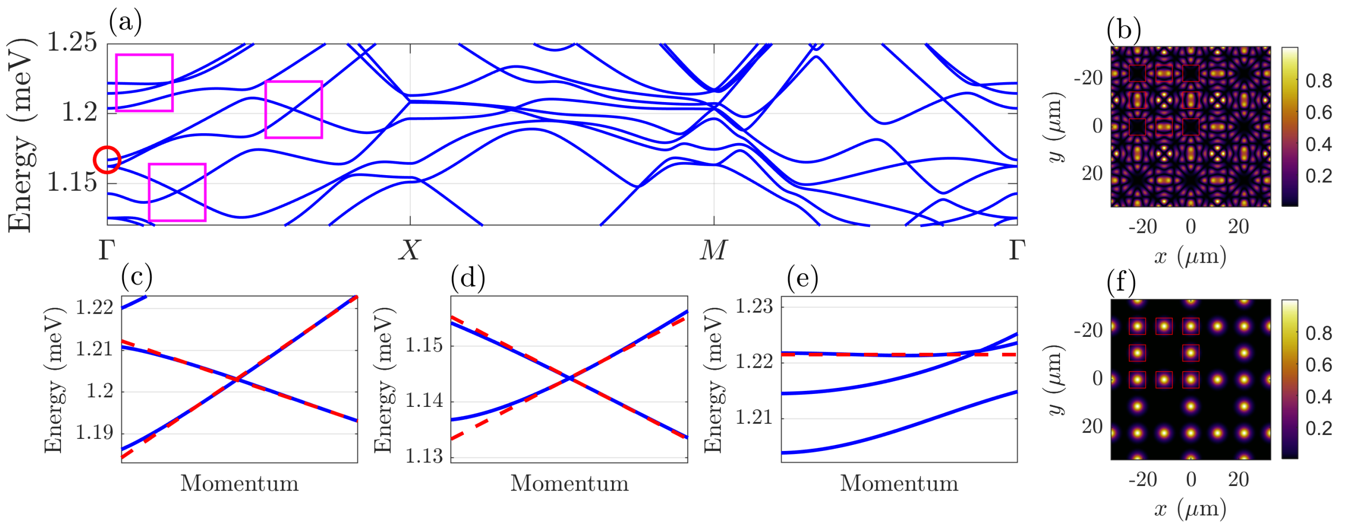

Supplementary Figure 8. **Calculated polariton bands using Bloch's theorem in the Lieb scatterer lattice.** (a) Calculated bands using Eq. (7) for a Lieb lattice of Gaussians, similar to Fig. 6 and Fig. 2. (b) Calculated Bloch state intensity $|u_{n_c,\mathbf{q}=0}(\mathbf{r})|^2$ belonging to the band marked by the red circle in (a) and the condensate real space PL from Fig. 2(b) in the main text. The arrangement of the Gaussian pumps is indicated by the red squares. (c-e) Zoomed in regions of the dispersion showing exotic points where the dispersion becomes practically linear (massless polaritons) or flat (heavy polaritons). (f) Potential profile $V(\mathbf{r})$ (normalised) shaped into a Lieb scatterer lattice of Gaussians with a lattice constant $D = 22.3$ μm and $V_0 = 1.25$ meV.

We can diagonalise Eq. (7) by expressing the Gaussian potential as a Fourier series in the basis of the reciprocal lattice vectors defined through $\mathbf{G}\cdot\mathbf{D} = 2\pi n$ for $n \in \mathbb{Z}$,

$$V(\mathbf{r}) = \sum_G V_G e^{i\mathbf{G}\cdot\mathbf{r}}. \tag{8}$$

Supplementary Fig. 8a shows a portion of the calculated band structure for a Lieb arrangement of the Gaussian spots (see Supplementary Fig. 8f) with a lattice constant of $D = 22.3$ μm with some features of interest marked with magenta colored boxes. Supplementary Fig. 8b shows the real space intensity of the Bloch state $|u_{n_c,\mathbf{q}=0}|^2$, marked by red circle corresponding to the experimentally observed pattern shown in Supplementary Fig. 2. A portion of the Gaussian pump locations are marked by red squares in both Supplementary Figs. 8b and 8f for clarity. In Supplementary Fig. 8a we observe regions in the band structure with almost linear- and flat-like bands (magenta squares zoomed in Supplementary Figs. 8c-e). Although such exotic points were not experimentally investigated in this study, their theoretical prediction enhances the versatility and importance of the investigated scatterer Lieb lattice. Even exciting polaritons into such points via non-resonant methods is infeasible they can still be excited using resonant lasers. This can potentially open up investigation into nonlinear transport properties of nearly massless or slow-light polaritons.

We also observe that the calculated Bloch state shown in Supplementary Fig. 8b shows complete destructive interference at sublattice B whereas in the experimentally measured spatial PL in Supplementary Fig. 2b this destructive interference is not complete. This could mean the the condensate is populating more than one Bloch state of the lattice since Supplementary Fig. 8a clearly shows that multiple bands exist within a narrow energy range (i.e., many bands can be within the gain bandwidth of the lattice). The time-integrated measurements of our experiment would then instead show a weighted average over these multiple Bloch states which explains the discrepancy between linear Bloch's theory and our measurements. In order to get better agreement between measurements and theory, we introduce in the next section a generalised Gross-Pitaevskii model which describes the polarion dynamics above condensation threshold (i.e., in the nonlinear regime).

SUPPLEMENTARY NOTE 4: GENERALISED GROSS-PITAEVSKII SIMULATIONS

We simulate the dynamics of the polariton condensate in the parabolic regime of the lower polariton branch using the two-dimensional driven-dissipative (i.e., generalised) Gross-Pitaevskii model[3] written for the condensate wavefunction,

$\Psi(\mathbf{r}, t)$, and a rate equation for the exciton reservoir supplying particles into the condensate, $n(\mathbf{r}, t)$:

$$i\frac{\partial \Psi}{\partial t} = \left[\frac{-\hbar \nabla^2}{2m} + \alpha|\Psi|^2 - \frac{i\gamma}{2} + \left(g + \frac{iR}{2}\right) n + GP(\mathbf{r})\right] \Psi + i\mathfrak{R}(\Psi), \tag{9}$$

$$\frac{\partial n}{\partial t} = -(\Gamma_R + R|\Psi|^2)n + P(\mathbf{r}). \tag{10}$$

Here, m is the polariton effective mass, α is the polariton-polariton interaction strength, R is the scattering rate of reservoir excitons into the condensate, γ is the rate of polariton losses through the cavity mirrors, Γ_R is the decay rate of the reservoir excitons, g and G are the interaction strengths of polaritons with the exciton reservoir feeding the condensate (i.e., so-called *bottleneck* or *active* excitons), and high momentum photoexcited excitons $P(\mathbf{r})$ (proportional to the laser profile and power) that do not scatter into the condensate, respectively. The final term $\mathfrak{R}(\Psi)$ is an energy relaxation term[4,5] that assists ground state condensation and is taken proportional to the background density of bottleneck excitons,

$$\mathfrak{R}(\Psi) = (\lambda n)^\xi \left[\frac{\hbar \nabla^2}{2m} + \mu(\mathbf{r}, t)\right] \Psi. \tag{11}$$

Here, λ denotes the energy relaxation efficiency, ξ is a fitting parameter, and $\mu(\mathbf{r}, t)$ is the particle conserving local effective chemical potential of the condensate.

To take into account to finite diffusion of the reservoir excitons $n(\mathbf{r}, t)$ away from the laser pump spots we approximate the reservoir driving profile with a Lorentzian,

$$P(\mathbf{r}) = P_0 \sum_{n=1}^{N} \frac{L^2}{L^2 + (x - x_n)^2 + (y - y_n)^2}. \tag{12}$$

Here, N is the number of pumps in question and x_n, y_n are their coordinates, $2L = 2$ μm denotes the full width at half maximum of the reservoir driving field and the value P_0 denotes the laser power density. We point out that the band features previously analysed in Sec. using lattices made up of arrranged Gaussian spots are not qualitatively changed here. While our model aims at being as simple as possible to produce the observed effects, more accurate simulations could possibly be achieved by introducing explicitly an exciton diffusion term into Eq. (10)[6] and stochastic noise[7]. The lower polariton mass and lifetime are taken corresponding to the properties of our cavity: $m = 0.32$ meV ps^2 μm^{-2}, and $\gamma = 1/5.5$ ps^{-1}. The remaining parameters are taken similar to those used in previous works: $\hbar\alpha = 3.3$ μeV μm^2, $g = \alpha$, $G = 2g/\Gamma_R$, $R = 0.8\alpha$, $\Gamma_R = 2\gamma$, $\xi = 1/4$, and $\lambda^\xi = 0.004$ μm^2.

In each simulation we apply stochastic initial conditions to $\Psi(\mathbf{r}, t = 0)$ and run the simulation much longer than the characteristic timescales of the condensate dynamics. This typically corresponds to simulation times longer than 10 ns. The results of such simulations are presented in the insets of Figs. 3a-c and 3e in the main text where we have plotted the time-integrated (averaged) condensate density $\langle|\Psi(\mathbf{r}, t)|^2\rangle$ at the same lattice constants as in experiment for pump powers P_0 chosen to give the best match. The single exception is the data presented in the inset in Fig. 3d which was not obtained from Gross-Pitaevskii simulations. This was due to the lack of the simulated condensate stabilising in a state similar to the one observed in experiment for the chosen parameters. It is important to stress that this does not preclude such a condensed state existing in stable form in simulation for other parameters. However, we are able to find a good match using Bloch's theorem [see Eq. (7)] where we plot in Fig. 3d the state $|\Psi(\mathbf{r})|^2 = |u_{3,\mathbf{q}_X}(\mathbf{r})|^2 + |u_{3,\mathbf{q}_{X'}}(\mathbf{r})|^2$ with $\mathbf{q}_X = (0, \pi/D)$ and $\mathbf{q}_{X'} = (\pi/D, 0)$ corresponding to the edges of the Brillouin zone.

In Fig. 3m in the main manuscript we qualitatively produce the experimental observation of gain guided condensates collapsing into trapped condensates at high power and small lattice constant through Gross-Pitaevskii simulations. Some discrepancy can be observed between simulation and experiment at low powers and small lattice constants where the simulation overestimates the gain guided polaritons. This discrepancy can appear from the lack of stochastic treatment (i.e., Wiener noise) in our equations which would smear out the simulated condensate PL at low powers close to threshold.

SUPPLEMENTARY NOTE 5: FINITE P-FLATBAND CURVATURE

As shown in Fig. 4i and 4j in the main manuscript, there exist residual finite band curvature in the P-flatbands of the inverse optical Lieb lattice. As mentioned in the text of of main manuscript the dispersion (curvature) of the flatband appears around the M point and is ≈ 5 µeV different in energy from both the Γ or the X points (see Supplementary Fig. 9b). Interestingly, the curvature of one flatband corresponds to lattice particles of negative effective mass and the

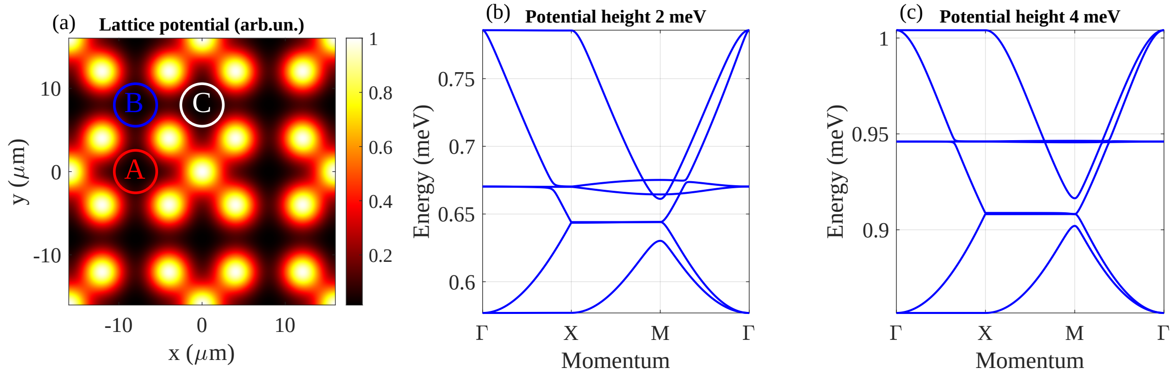

Supplementary Figure 9. **Effects of the inverse optical Lieb lattice potential height on the P-bands.** (a) Four unit cells of the Pump induced potential landscape used in Bloch's analysis. (b) Calculated band structure for peak potential height of 2 meV. (c) Calculated band structure for peak potential height of 4 meV. The polariton mass is set to $m = 0.32$ meV ps^2 μm^{-2}.

other to positive effective mass. Intuitively, the nonequilibrium nature and energy relaxation of polaritons triggers condensation into the minima of the lower flatband. The source of this curvature is attributed to the finite potential depth and the Gaussian shape of the excitation beams (see Supplementary Fig. 9a) which leads to deviation from the perfectly dispersionless bands predicted by the tight binding theorem. By increasing the depth of the potential minima (or conversely, the height of the potential maxima) in the lattice the flatband curvature is reduced as can be seen in Supplementary Fig. 9c. The flatbands in Supplementary Fig. 9c are also blueshifted (with respect to the other P bands) which comes from a slightly increased energy splitting between the P_x and P_y orbitals at sublattices A and C due to the Gaussian construction of our lattice.

SUPPLEMENTARY NOTE 6: TIGHT BINDING TREATMENT

Here, we show qualitatively why condensation preferably occurs into a flatband P-orbital based by considering a tight-binding treatment of the P-orbital states. We consider only nearest neighbor coupling and negligible mixing with neighboring S- and D-orbitals. Being a scalar problem, we also do not consider the polarization degree of freedom of the polaritons. The bulk Hamiltonian in the basis of single particle, lattice site, creation and annihilation operators can be written,

$$\begin{aligned}\hat{H} = \sum_{n,m} \Big[&(\hat{b}^{(x)}_{n,m})^\dagger [J_2(\hat{a}^{(x)}_{n,m} + \hat{a}^{(x)}_{n-1,m}) - J_1(\hat{c}^{(x)}_{n,m} + \hat{c}^{(x)}_{n,m-1})] + \text{h.c.} \\ &+ (\hat{b}^{(y)}_{n,m})^\dagger [J_1(\hat{a}^{(y)}_{n,m} + \hat{a}^{(y)}_{n-1,m}) - J_2(\hat{c}^{(y)}_{n,m} + \hat{c}^{(y)}_{n,m-1})] + \text{h.c.} \\ &+ \epsilon_{a,x}(\hat{a}^{(x)}_{n,m})^\dagger \hat{a}^{(x)}_{n,m} + \epsilon_{b,x}(\hat{b}^{(x)}_{n,m})^\dagger \hat{b}^{(x)}_{n,m} + \epsilon_{c,x}(\hat{c}^{(x)}_{n,m})^\dagger \hat{c}^{(x)}_{n,m} \\ &+ \epsilon_{a,y}(\hat{a}^{(y)}_{n,m})^\dagger \hat{a}^{(y)}_{n,m} + \epsilon_{b,y}(\hat{b}^{(y)}_{n,m})^\dagger \hat{b}^{(y)}_{n,m} + \epsilon_{c,y}(\hat{c}^{(y)}_{n,m})^\dagger \hat{c}^{(y)}_{n,m} \Big]. \end{aligned} \tag{13}$$

Here, $\hat{a}^{(x,y)}_{n,m}, \hat{b}^{(x,y)}_{n,m}, \hat{c}^{(x,y)}_{n,m}$ denote the annihilation operators at sublattices A, B, C respectively [see Supplementary Fig. 4], (n, m) denotes the site index of the corresponding sublattice, and (x, y) P-orbital lobe alignment along the two orthogonal coordinates x, y of the lattice. The operators obey bosonic commutation relation $[\hat{a}^{(\alpha)}_{n,m}, (\hat{a}^{(\beta)}_{n',m'})^\dagger] = \delta_{\alpha,\beta}\delta_{n,n'}\delta_{m,m'}$ and $[\hat{a}^{(\alpha)}_{n,m}, \hat{a}^{(\beta)}_{n',m'}] = 0$. The coefficients $J_{1,2} \in \mathbb{C}$ denote the polariton non-Hermitian hopping amplitudes between P-orbitals with orbital axes parallel (σ-bond) and perpendicular (π-bond) to the bond axis respectively. The coefficients $\epsilon_{N,\alpha}$ denote on-site energy of the two distinct P-orbitals at each sublattice.

Performing the standard Fourier transform of the single particle operators one can transform the problem from the

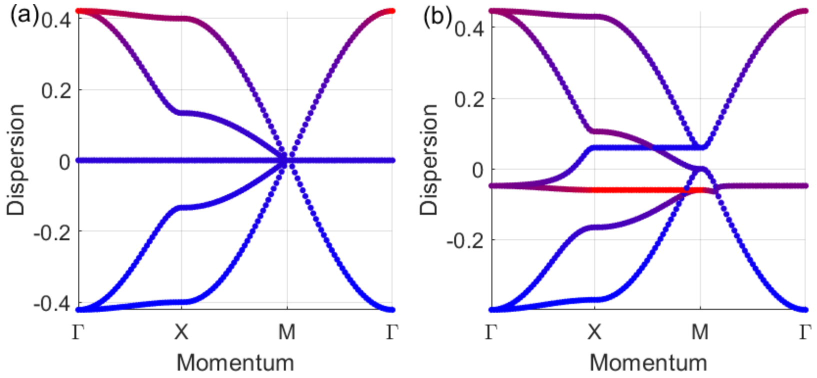

Supplementary Figure 10. **Calculated P-orbital bands using tight binding theorem.** Blue to red colorscale shows energies of small and large imaginary part (gain) respectively. (a) All diagonal elements $\epsilon_{N,\alpha} = 0$ resulting in highest gain at the top of the conduction P-bands. (b) Non-zero diagonal elements introducing splitting between P_x and P_y orbitals at sublattices A and C results in gain moving towards the flatband.

basis of lattice sites (n, m) into the basis of crystal momentum $\mathbf{q} = (q_x, q_y)$.

$$\frac{\hat{H}_{\mathbf{q}}}{2} = \begin{pmatrix} \epsilon_{a,x} & J_2 e^{i\frac{q_y}{2}} \cos\left(\frac{q_y}{2}\right) & 0 & & & \\ J_2 e^{-i\frac{q_y}{2}} \cos\left(\frac{q_y}{2}\right) & \epsilon_{b,x} & J_1 e^{i\frac{q_x}{2}} \cos\left(\frac{q_x}{2}\right) & & & \\ 0 & J_1 e^{-i\frac{q_x}{2}} \cos\left(\frac{q_x}{2}\right) & \epsilon_{c,x} & & & \\ & & & \epsilon_{a,y} & J_1 e^{i\frac{q_y}{2}} \cos\left(\frac{q_y}{2}\right) & 0 \\ & & & J_1 e^{-i\frac{q_y}{2}} \cos\left(\frac{q_y}{2}\right) & \epsilon_{b,y} & J_2 e^{i\frac{q_x}{2}} \cos\left(\frac{q_x}{2}\right) \\ & & & 0 & J_2 e^{-i\frac{q_x}{2}} \cos\left(\frac{q_x}{2}\right) & \epsilon_{c,y} \end{pmatrix}. \tag{14}$$

Setting all diagonal terms to zero corresponds to all sites being in resonance and with equal on-site gain. In Supplementary Fig. 10a we plot the dispersion from Eq. (14) for $J_1 = 0.4 + i0.04$ and $J_2 = J_1/3$. The blue-red colorscale indicates energies with low and high particle gain (imaginary part of the energy), respectively. In this case it shows that polaritons will survive the longest around the Γ point in the upper bands.

On the other hand, if the x and y P-orbitals become split in both real energy and gain at their respective sublattice sites then the dispersion dramatically changes. In Supplementary Fig. 10b we show the calculated energies again for $J_1 = 0.4 + i0.04$ and $J_2 = J_1/3$ but with x and y P-orbitals split at sublattices A and C. This is modeled by setting $\epsilon_{a,x} = \epsilon_{c,y} = -\epsilon_0 + i\kappa$, $\epsilon_{a,y} = \epsilon_{c,x} = \epsilon_0 - i\kappa$, and $\epsilon_{b,x} = \epsilon_{b,y} = 0$ where $\epsilon_0, \kappa > 0$. Such splitting arises from the slightly elliptically shaped A and C sublattice sites [see Supplementary Fig. 7a]. Physically, κ captures the increased gain of the lower energy state due to greater overlap with the gain region from the Gaussian pumps. In contrast to Supplementary Fig. 10a the flatband between the X and the M points in Supplementary Fig. 10b has now obtained the highest gain due to this non-Hermitian splitting between the x, y P-orbitals in the A and C sublattices.

* P.Lagoudakis@skoltech.ru

[1] Kavokin, A. V., Baumberg, J. J., Malpuech, G. & Laussy, F. P. *Microcavities*, vol. 21 (Oxford university press, 2017).

[2] Cilibrizzi, P. *et al.* Polariton condensation in a strain-compensated planar microcavity with ingaas quantum wells. *Applied Physics Letters* **105**, 191118 (2014). URL https://doi.org/10.1063/1.4901814.

[3] Wouters, M. & Carusotto, I. Excitations in a nonequilibrium bose-einstein condensate of exciton polaritons. *Phys. Rev. Lett.* **99**, 140402 (2007). URL https://link.aps.org/doi/10.1103/PhysRevLett.99.140402.

[4] Wouters, M., Liew, T. C. H. & Savona, V. Energy relaxation in one-dimensional polariton condensates. *Phys. Rev. B* **82**, 245315 (2010). URL https://link.aps.org/doi/10.1103/PhysRevB.82.245315.

[5] Askitopoulos, A. *et al.* Robust platform for engineering pure-quantum-state transitions in polariton condensates. *Phys. Rev. B* **92**, 035305 (2015). URL https://link.aps.org/doi/10.1103/PhysRevB.92.035305.

[6] Tosi, G. *et al.* Sculpting oscillators with light within a nonlinear quantum fluid. *Nature Physics* **8**, 190–194 (2012). URL https://doi.org/10.1038/nphys2182.

[7] Töpfer, J. D. *et al.* Engineering spatial coherence in lattices of polariton condensates. *Optica* **8**, 106–113 (2021). URL http://www.osapublishing.org/optica/abstract.cfm?URI=optica-8-1-106.
