# Supplementary material for: Quantum fluids of light in all-optical scatterer lattices
Source: Nat Commun. 2021 Sep 22;12:5571. doi: 10.1038/s41467-021-25845-4 (PMC8458361; doi:10.1038/s41467-021-25845-4)
Supplement: Supplementary file 3 — Description of Additional Supplementary Files [file 41467_2021_25845_MOESM3_ESM.pdf]

Description of Supplementary Files

Supplementary PDF:

Supporting experimental and theoretical data/information for the content of the main manuscript.

Supplementary Movie 1:

Energy tomography of an optical scatterer Lieb lattice of polariton condensates with lattice constant $D \approx 20.3$ µm. The video shows the experimentally recorded spectrally-resolved momentum space photoluminescence with k_x and k_y as horizontal and vertical axis, respectively. The movie starts at high energy $E = 1.44967$ eV and ends at low energy $E = 1.4455$ eV.
